# Temporal alignment of electrocorticographic recordings for upper limb movement

**DOI:** 10.3389/fnins.2014.00431

**Published:** 2015-01-13

**Authors:** Omid Talakoub, Milos R. Popovic, Jessie Navaro, Clement Hamani, Erich T. Fonoff, Willy Wong

**Affiliations:** ^1^Department of Electrical and Computer Engineering, University of TorontoToronto, ON, Canada; ^2^Institute of Biomaterials and Biomedical Engineering, University of TorontoToronto, ON, Canada; ^3^Rehabilitation Engineering Laboratory, Toronto Rehabilitation Institute, University Health NetworkToronto, ON, Canada; ^4^Division of Functional Neurosurgery of Institute of Psychiatry, Department of Neurology, University of Sao Paulo Medical SchoolSao Paulo, Brazil; ^5^Division of Neurosurgery, Toronto Western Hospital, University of TorontoToronto, ON, Canada

**Keywords:** electrocorticography, ECoG, arm movement, dynamic time warping, kinematics, movement classification

## Abstract

The detection of movement-related components of the brain activity is useful in the design of brain-machine interfaces. A common approach is to classify the brain activity into a number of templates or states. To find these templates, the neural responses are averaged over each movement task. For averaging to be effective, one must assume that the neural components occur at identical times over repeated trials. However, complex arm movements such as reaching and grasping are prone to cross-trial variability due to the way movements are performed. Typically initiation time, duration of movement and movement speed are variable even as a subject tries to reproduce the same task identically across trials. Therefore, movement-related neural activity will tend to occur at different times across the trials. Due to this mismatch, the averaging of neural activity will not bring into salience movement-related components. To address this problem, we present a method of alignment that accounts for the variabilities in the way the movements are conducted. In this study, arm speed was used to align neural activity. Four subjects had electrocorticographic (ECoG) electrodes implanted over their primary motor cortex and were asked to perform reaching and retrieving tasks using the upper limb contralateral to the site of electrode implantation. The arm speeds were aligned using a non-linear transformation of the temporal axes resulting in average spectrograms with superior visualization of movement-related neural activity when compared to averaging without alignment.

## Introduction

The challenge for a brain-machine interface (BMI) is to decode user intent and to transform neural signals into signals which drive an external device like a prosthetic arm. This technology holds enormous potential as an assistive device for individuals with limited ability to perform voluntary movements. Examples of populations that may benefit from this technology include individuals with stroke, advanced stages of amyotrophic lateral sclerosis (Kübler et al., 2001, 2005; Bai et al., 2008; Nijboer et al., 2008), severe cerebral palsy (Pfurtscheller et al., 2000), and high level cervical spinal cord injury (Wolpaw et al., 2000). However, the construction of a BMI platform is predicated first on the ability to identify the salient neural activity associated with upper limb movement (Pfurtscheller et al., 2003; Leuthardt et al., 2004; Foffani et al., 2005; Rickert, 2005; Chin et al., 2007; Schalk et al., 2007; Bai et al., 2008; Ball et al., 2008, 2009; Miller and Ojemann, 2009; Tzagarakis et al., 2010; Zhuang et al., 2010).

One challenge in uncovering the salient neural activity associated with movement is the variability of neural activities and the low signal-to-noise ratio (SNR) that is commonly found for electrophysiological recordings. While gamma power tends to be higher in ECoG recordings, not all frequency bands have high SNR. Moreover, the clarity of gamma activity may depend on individual factors including placement of electrodes. As such developing techniques for applications with low SNR is crucial for uncovering movement-related activities. Typically, noise and variability is dealt with by averaging a large number of repeated trials. To do this, however, one must assume that the neural activity is time-locked to motor-specific events like movement onset. While the evoked brain activity from external stimuli or highly constrained motor tasks can be thought of as being identical on a trial-by-trial basis, this is certainly not true when a subject is performing a complex movement task like reaching. Due to the difficulty in constraining the movement of the arm, it would be unwise to simply take all trials and average them. Instead, we propose a method of realignment through a non-linear transformation in time. This transformation accounts for differences in movement initiation, arm speeds, and movement durations. After alignment, we expect the neural activities to occur at near identical times and that the related neural activities can now be more effectively brought into salience through averaging.

Other approaches seek to decode movement intention in real-time without use of template matching of templates, (e.g., Wang et al., 2012). Our study is more modest in that our primary focus is with movement classification. This paper concerns the initial application of time warping for movement co-registration. We will discuss the application to classification later in the Discussion Section.

## Background

Averaging of neural activities is a practice standard in electrophysiology. For example, event-related potentials (ERP) are time-averaged brain responses to a sensory or motor event. They are simple to calculate and are widely used in clinical neurology for diagnostic purposes. The ERP's show mostly low frequency neural activity since the high frequency components tend to be off-phase from trial-to-trial thereby canceling out through averaging. An alternative to averaging in the time-domain is to average their time-frequency representations. The time-frequency representation of a signal (e.g., a spectrogram) details the spectral density of the signal as a function of time. Similar to averaging over time, averaging over time-frequency space can aid in highlighting the time-dependent spectral density of related neural activities. A triggered, synchronized decrease in band power is known as event-related desynchronization (ERD) and a corresponding increase is known as an event-related synchronization (ERS). These events are measured with respect to a chosen baseline in activity. The baseline is typically set to the rest state.

The electrical activity of the brain can be recorded using a number of different methods including (1) electroencephalography (EEG) where electrodes are placed on the scalp, and (2) single neuron or neuronal ensemble recordings obtained through micro-electrodes placed intra-cortically in proximity of target neurons. Electrocorticography (ECoG) is a method of recording the electrical activities of the brain using macro-electrodes placed surgically over or under the dura. In this study, ECoG contacts were implanted over the dura. The signals obtained using these electrodes generally have higher SNR, a wider bandwidth, and higher spatial resolution when compared to electroencephalography recordings (Schalk et al., 2007; Schalk and Leuthardt, 2011). Additionally, this technology is less invasive than intracortical recordings since the electrodes do not penetrate the brain tissue. In this study, we processed the activity of the motor cortex recorded from two ECoG contacts in a bipolar arrangement.

Typically, neuromotor activities are aligned to a “go” signal (Ball et al., 2009; Reddy et al., 2009; Tzagarakis et al., 2010), to movement onset (Sergio and Kalaska, 1998; Moran and Schwartz, 1999; Rickert, 2005; Miller and Ojemann, 2009) or to movement termination (Jurkiewicz et al., 2006). The alignment strategy is determined in part by the experimental paradigm and by what questions the experimenters would like to answer from their data. For example, activities aligned to the “go” signal would allow for the study of movement preparation. The problem with event-based alignment is that this does not guarantee that the remainder of the trial is similarly aligned. If we were interested also in, say, movement termination, the trials would then need to be realigned to the end point of movement cycle. To eliminate repeated analyses, we instead introduce a new method of alignment involving a non-linear transformation of time. We believe that this transformation can account for the temporal differences in the way a motor task is conducted.

Temporal alignment of biological signals is not new; for example, such techniques have been employed extensively as part of automatic speech recognition algorithms. Non-linear time warping has also been used to align physiological signals (Munhall et al., 2004) as well as neural signals (Picton et al., 1988; Wang et al., 2001; Casarotto et al., 2003; Cho et al., 2004; Karamzadeh et al., 2013). More recently, Pasley et al. (2012) explored the reconstruction of auditory speech features using ECoG recordings. Their work is quite similar to ours as they use dynamic time warping to realign a transformed representation of the neural signal. We have used warping in the context of aligning movement-related neural activity.

Dynamic time warping (Sakoe and Chiba, 1978) is a graph-based approach to calculate the time transform required to align two signals. The time transformation (or time registration path) is calculated by minimizing a cost function which measures the similarities between the time instances of two signals. Dynamic time warping has been used previously to align sensory evoked responses (Picton et al., 1988; Wang et al., 2001; Casarotto et al., 2003). Picton et al. used dynamic time warping to align the brain-stem auditory evoked response showing improvement in the visualization of components over simple averaging (Picton et al., 1988). In a similar study, Wang et al. showed that the amplitude of the derived visual ERP can be increased by up to 76% after realignment using dynamic time warping (Wang et al., 2001). Casarotto et al. used dynamic time warping to quantify the latencies between the ERPs obtained from normal and dyslexic children (Casarotto et al., 2003). All of these studies show that a simple shift or linear scaling of time is not sufficient to align evoked components.

Earlier studies of movement-related neural activity rely on simple or constrained movements to avoid problems with averaging of trials. However, this is not possible for a study involving complex movements due to the difficulty of constraining the movement of a participant to allow for careful control of arm kinematics. Instead, we introduce this new method of non-linear alignment to correct for the temporal mismatch.

## Materials and methods

### Participants

Four male participants were recruited from Functional Neurosurgery Clinic at the Hospital das Clínicas of University of São Paulo. Subject 1 was 51 years old, Subject 2 was 48 years old, Subject 3 was 42 years old, and Subject 4 was 58 years old. All participants were implanted with unilateral epidural quadripolar electrodes over the motor cortex for the treatment of chronic pain. After the insertion of the electrodes, patients had their systems externalized for 6 days for the selection of optimal stimulation parameters (polarity, amplitude, frequency, duration, etc.). Once these were chosen, the electrodes were connected to an implantable pulse generator during a second surgical procedure. The experiment took place over the 6 days during which the electrode leads were externalized. The study was approved by the University of Sao Paulo research ethics board, and all participants signed a letter of consent prior to taking part in the experiments.

### Electrodes and post-operative recordings

The placement and choice of number of ECoG contacts were dictated by the clinical requirements unrelated to the purpose and consideration of this study.

**Figure 1:**
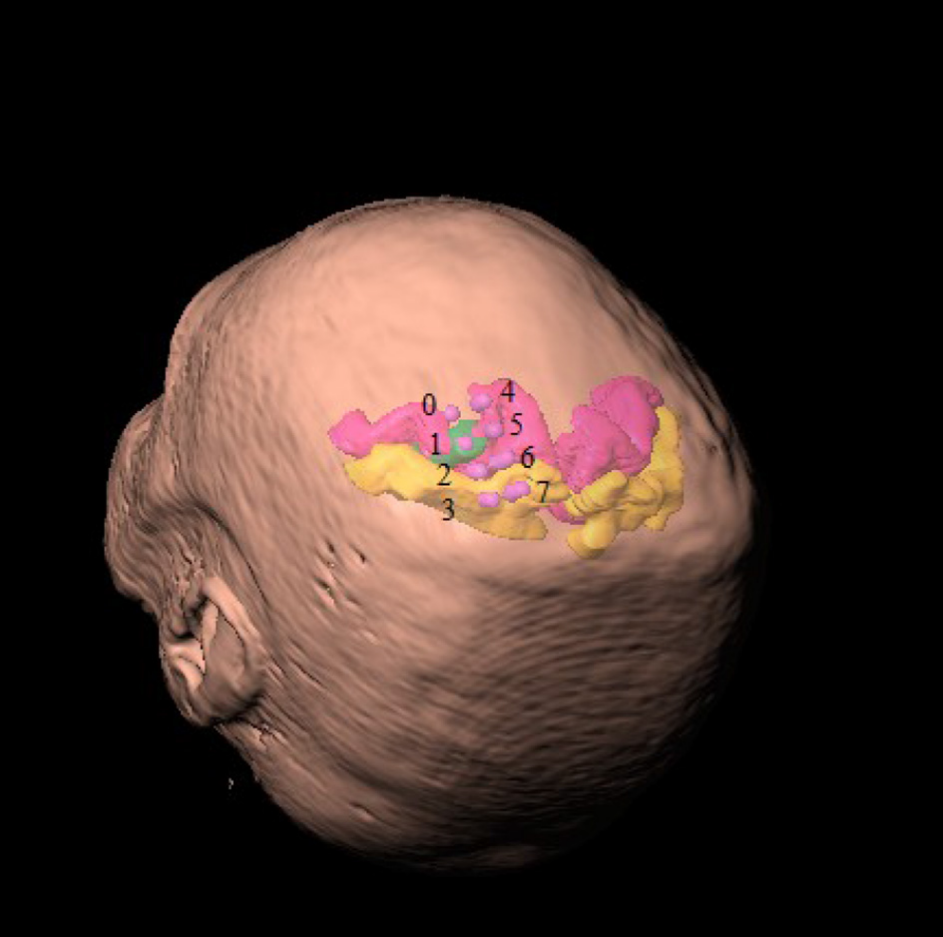
**Location of implanted ECoG contacts with respect to head representation**. Contacts of the first strip of electrodes are labeled 0–3 from distal to proximal and contacts of the second strip are similarly indexed 4–7. Primary motor cortex is colored in velvet and the primary sensory cortex is colored in amber. The area associated with the hand representation is marked in green.

The participants were implanted with two quadripolar epidural electrodes Lamitrode 3240 (St. Jude Medical Inc., U.S.A.). Each strip consists of a single row of four platinum discs that were 4 mm in diameter and had center-to-center distance of 10 mm. The electrodes were embedded in a silicon membrane. All participants were implanted with two electrode strips. The electrode strips were placed over the premotor, primary motor, and sensory cortices associated with the upper extremity representations. The first strip was placed on the cortices such that the second contact (electrode #1) was over the primary motor cortex. The location of the electrode was confirmed using electrical stimulation and by observing muscle contractions of the contralateral upper limb. Stimulation parameters were: (i) pulse frequency 50 Hz, (ii) pulse duration 100 μs, (iii) monopolar pulses, and (iv) pulse amplitude 3–10 μA. Electrode contacts were numbered 0–3 from distal to proximal. Specifically, stimulation of contact #1 implanted over the motor cortex induced finger or wrist movements. Electrode contacts of the second strip were numbered 4–7 from distal to proximal. The second strip was placed dorsal to the first such that contact #5 (the second contact of the second strip) was positioned over the primary motor cortex and dorsal to contact #1. Figure 1 shows exemplary illustration of the location of implanted electrodes with respect to the head and the cortical area associated with the upper limb.

In addition to the ECoG measurements, electroencephalography (EEG) signals were recorded at the C3/C4, Cz, Fz, and FP1 locations according to the 10-20 electrode placement system. The purpose for recording EEG was to identify the trials contaminated with eye movement or muscle artifact and to reject the trials contaminated with these artifacts. They were not otherwise used in the analyses. Moreover, electromyography (EMG) signals were obtained from the wrist flexors, wrist extensors, biceps, and triceps. The EEG, ECoG, and EMG signals were recorded using a sampling frequency of 1200 Hz with a 16-channel g.USBamp biosignal acquisition device (g.tec, Graz, Austria). The recording device has a built-in anti-aliasing filter which is dependent on sampling frequency. The anti-aliasing filter is an 8th order digital Butterworth filter with pass-band frequency at 0.1–500 Hz (g.USBamp manual, 2011). The activity of the motor cortex was recorded from two ECoG contacts in bipolar arrangement (contacts #0 and #1). The choice of contact #1 was made because its placement was verified using stimulation of the contact to induce finger or wrist movement. Contact #0 was chosen as we required it to be adjacent to contact #1, and also to lie above the motor cortex which was verified using MRI.

### Experimental setup

The participants sat in a comfortable chair. The upper limb movements were recorded using a three dimensional (X, Y, Z) electromagnetic tracker, Fastrack (Polhemus Inc, U.S.A.) and a customized data acquisition software written in C. A motion sensor was placed over the dorsal aspect of the third metacarpal bone of the hand. The three-dimensional position of the sensor was recorded with sampling frequency of 40 Hz and was time stamped. The upper limb kinematics were recorded using the same computer that captured the ECoG, EEG, and EMG data. Thus the kinematic recordings were synchronized with the electrophysiological recordings.

### Experimental protocol

ECoG, EEG, and EMG signals were recorded while the participants performed a reaching task. The task was carried with the arm contralateral to the site of electrode implantation. The task was to reach a target placed 40 cm away from the chest which the participant could do comfortably. At the start of the task the participant had his or her hand in a resting position where the hand was placed on a pillow located on their lap. Under resting conditions EMG muscle activity was not observed. The participants received an auditory cue (“go” signal) to start the reaching task. After completing this, the participants were instructed to wait for few seconds before returning their hand to the initial resting place (retrieving task). The time between the end of the last trial and the cue signal of the next trial was randomized, and in the range of 8–10 s. Although the movements are voluntary, the reach task was triggered by an external “go” signal. As such they are not strictly self-paced in nature. However, the return motion was self-paced in that the subject had control as to when to initiate this movement.

Each participant performed at least 50 reaching tasks. Number of trials and averaged length of movements performed by each participant are shown in Table 1. Trials were extracted from ECoG recordings for offline analysis. A trial was defined as the period beginning 4 s prior to and ending 8 s after the onset of the reaching task. Movement onset itself was defined as the instance where arm speed exceeded a threshold of “0.5 cm/s” and was designated as *t* = 0.

**Table 1:** **Table summarizes mean and standard deviation of reaching and retrieval tasks as well as the pause between two tasks. Participant 3 did not pause between the tasks as was instructed**.

**Participant**	**Number of trials**	**Reaching duration (s)**	**Pause duration (s)**	**Retrieval duration (s)**
1	68	1.2 ± 0.3	1.3 ±0.4	1.0 ± 0.3
2	50	2.1 ± 0.7	2.7 ± 1.0	1.5 ± 0.6
3	71	1.0 ± 0.2	0.6 ± 0.28	1.0 ± 0.3
4	59	0.7 ± 0.2	2.8 ± 0.9	1.0 ± 0.4

### Analysis

The data were analyzed in the time-frequency domain using a *spectrogram*. A spectrogram gives the windowed short-time Fourier transform of a signal by describing the frequency content of a signal and how it changes over time. Signals were windowed in segments of 100 ms using a Hamming window. A Fourier transform was then computed for the windowed signal resulting in a spectrum with resolution of 1 Hz. The window was then shifted forward by 10 ms, and the procedure was repeated until the end of the epoch was reached. The resulting spectrogram consists of a matrix where each row represents the power spectrum of a windowed signal. Each column of this matrix represents the time series of power at a particular frequency. Event-related changes (ERD/ERS) were calculated by normalizing each column (frequency) by the baseline power. The baseline was defined as the average power between 1 and 2 s prior to movement onset.

Spectrograms are typically averaged without realignment along the time axis. This is the usual method of finding the spectral density of neural activity and how it changes with respect to movement. We call this method *conventional averaging*. In the second method, the epochs were warped in time according to the arm kinematics prior to averaging. One trial is chosen as the “gold-standard” or reference trial—all the other trials are then warped to this reference trial using a *time-registration path*. Given that warping of the signal in the time-domain distorts its spectral content, the time transformations were not applied to the raw ECoG recordings. Instead, warping was performed over the spectral densities such that the neural activity corresponding to the same arm velocity is identical across all trials. In this case, not only will movement onset and offset be aligned, but the neural activity at each time-point will correspond to the same arm speed/position. We call this method *time-warped averaging*.

### Realignment of the trials using dynamic time warping according to arm speed

**Figure 2:**
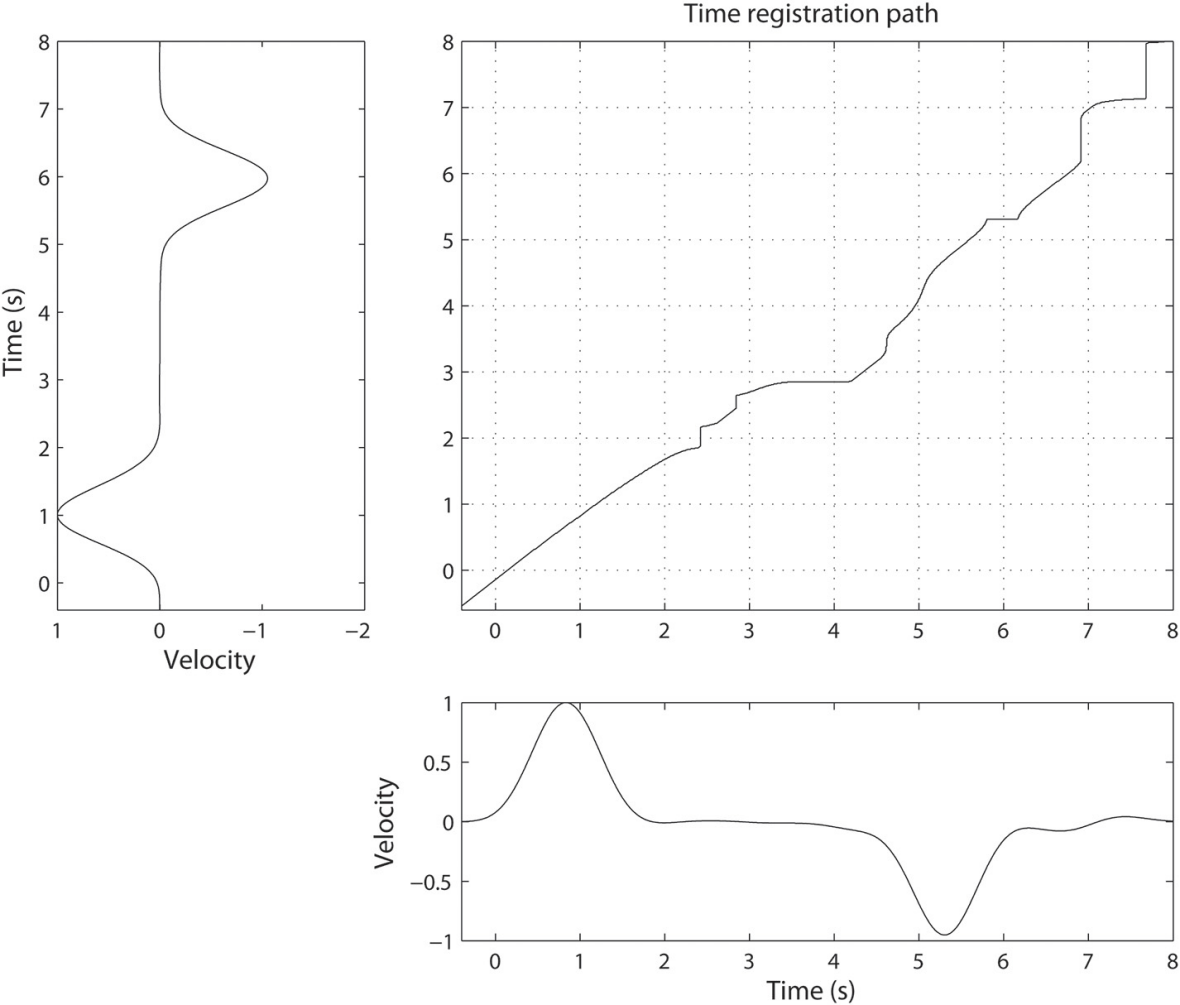
**Illustration of non-linear alignment or time-warping**. The time registration path defines how one time point in one trial maps to another time point in a different trial. The difference between two velocities becomes minimal when the time axis is transformed using the time-registration path.

Dynamic time warping algorithm was implemented using a symmetric step pattern with no constraint on the slope according to the method of (Sakoe and Chiba, 1978). Time-warping of ECoG signals was carried out over arm velocity such that the velocity profiles would be identical after alignment was complete. For simplicity, we warped along the X-axis (orthogonal to the chest surface) since it is the largest component over which movement took place. To determine the time registration path, we chose the Euclidean distance as a cost function and found the corresponding time points across the two time axes such that the Euclidean distance in arm velocities was minimized. Figure 2 shows an example of the registration path obtained by time-warping the two trials. This process aligns the time course of the movements (and therefore the spectral density of the associated neural activity) to a “gold standard.”

We performed statistical analyses to evaluate the efficacy of our approach. In this case, the reference or standard trial was permuted across all available trials. We compared the variability of the spectrograms when they were warped versus when they were not. The error obtained when the spectrograms are not warped comprises the null distribution. Error is defined as the RMS value calculated by a sum of square errors across all time-frequency cells. The error values resulted in two distributions and the distance between these two distributions provides a measure of the improvement due to warping. While non-parametric methods can be used in the analyses, we have found that in practice the log transform of the error resembles a normal-like distribution. Hence the *t*-test was used instead.

## Results

### Conventional averaging of ECoG signals

Overall, the time-frequency representation of the ECoG response shows a very distinct pattern of activity over the course of the movement. The power in the beta activity (12–30 Hz) is attenuated during the course of movement execution while the power in the gamma activity (65–140 Hz) is increased. These changes in power are statistically significant (*p* < 0.05, Kolmogorov–Smirnov test).

**Figure 3:**
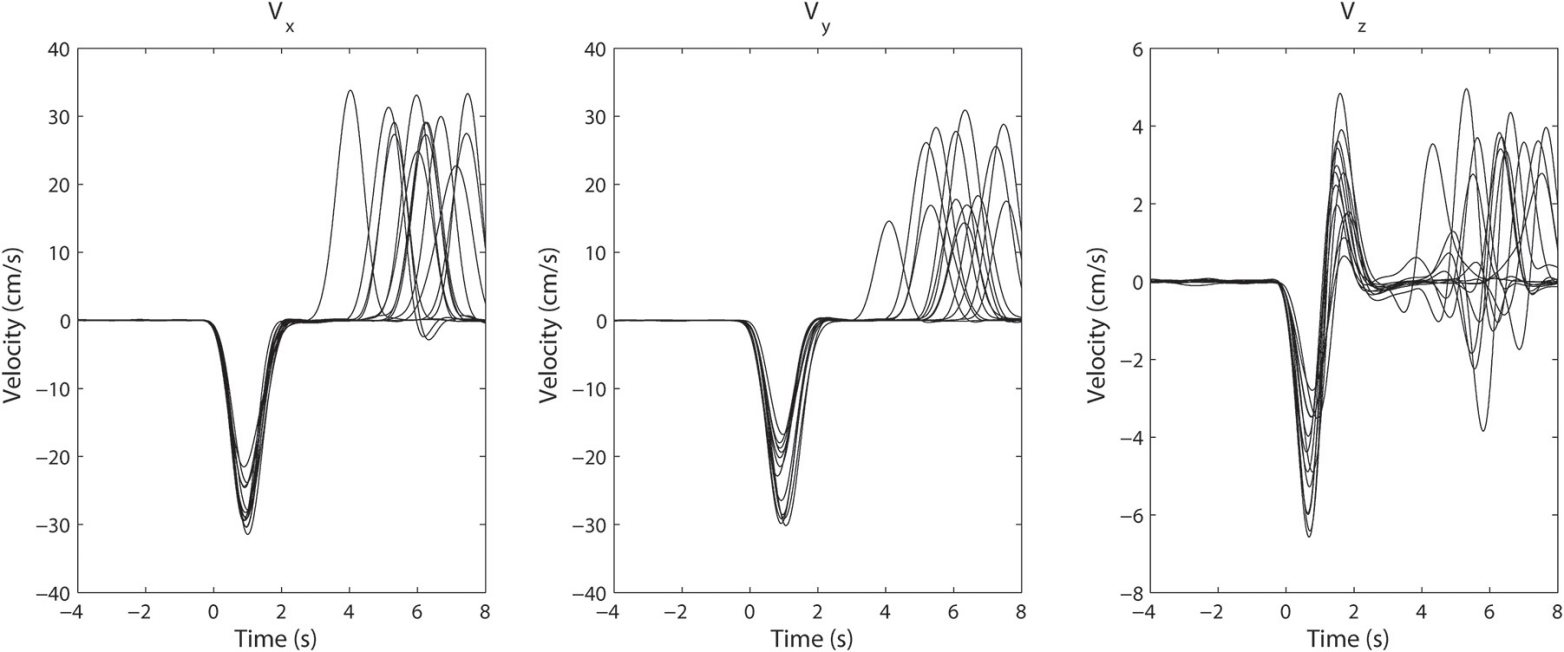
**Superimposed arm velocity for 25 trials when Subject 1 was reaching for a target**. The subject's arm returned to its initial location after a pause of several seconds having reached the target. The trials were aligned to the initial movement onset and are marked as *t* = 0. The variation in the duration of movement is clearly visible.

**Figure 4:**
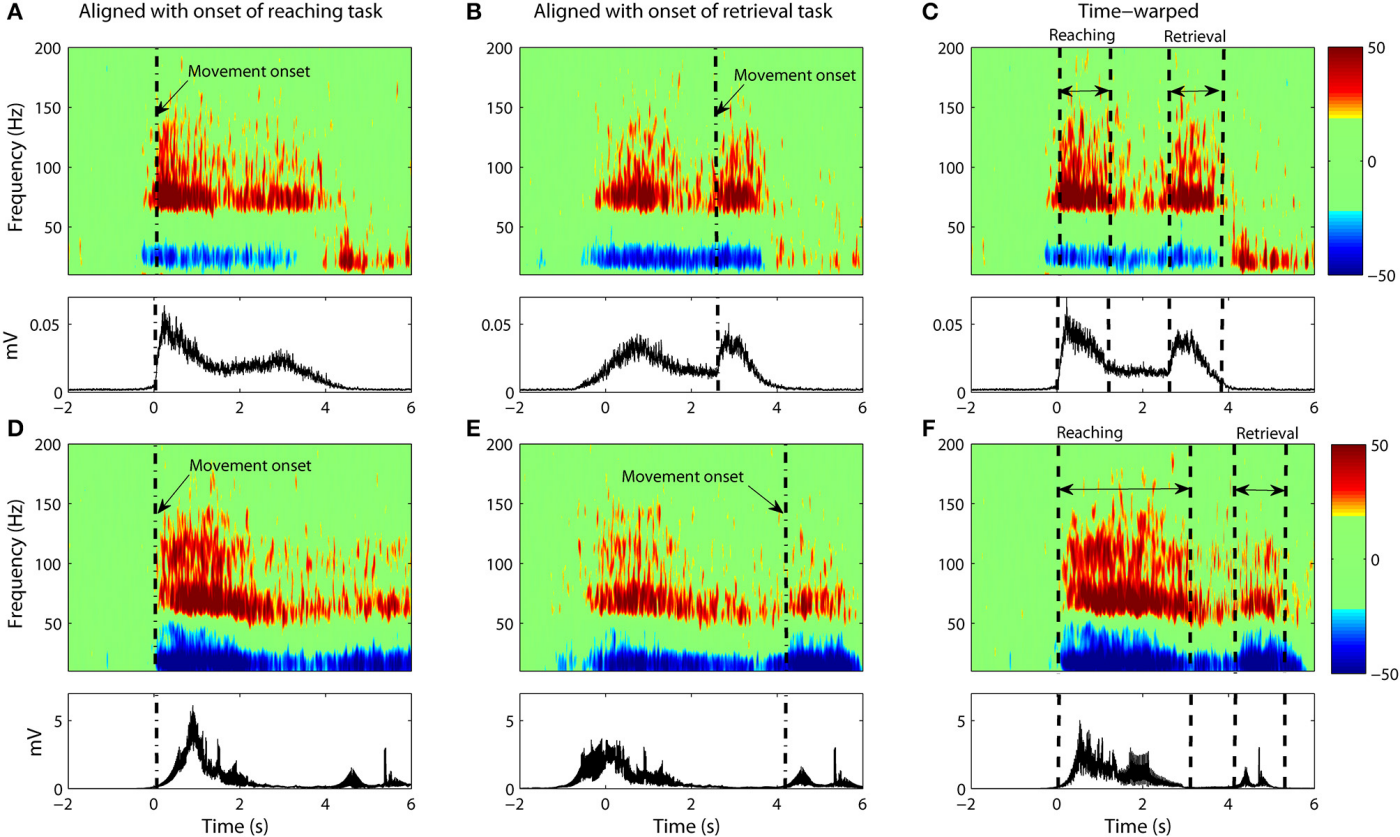
**(A–C)** Data from Subject #1. **(A)** The average spectrogram and the associated EMG activity as calculated through conventional averaging. Epochs were aligned with respect to onset of the reaching task with no time-warping used. Movement onset is denoted by dash dotted line (“-.”). The average spectrogram was normalized with respect to the baseline, which is defined as the power between 1 and 2 s prior to movement onset. **(B)** Same as **(A)** but with data aligned to movement onset of the retrieval task. **(C)** Same as **(A,B)** but with non-linear warping of the time axis prior to averaging. **(D–F)** Shows the same for Subject #2.

**Figure 5:**
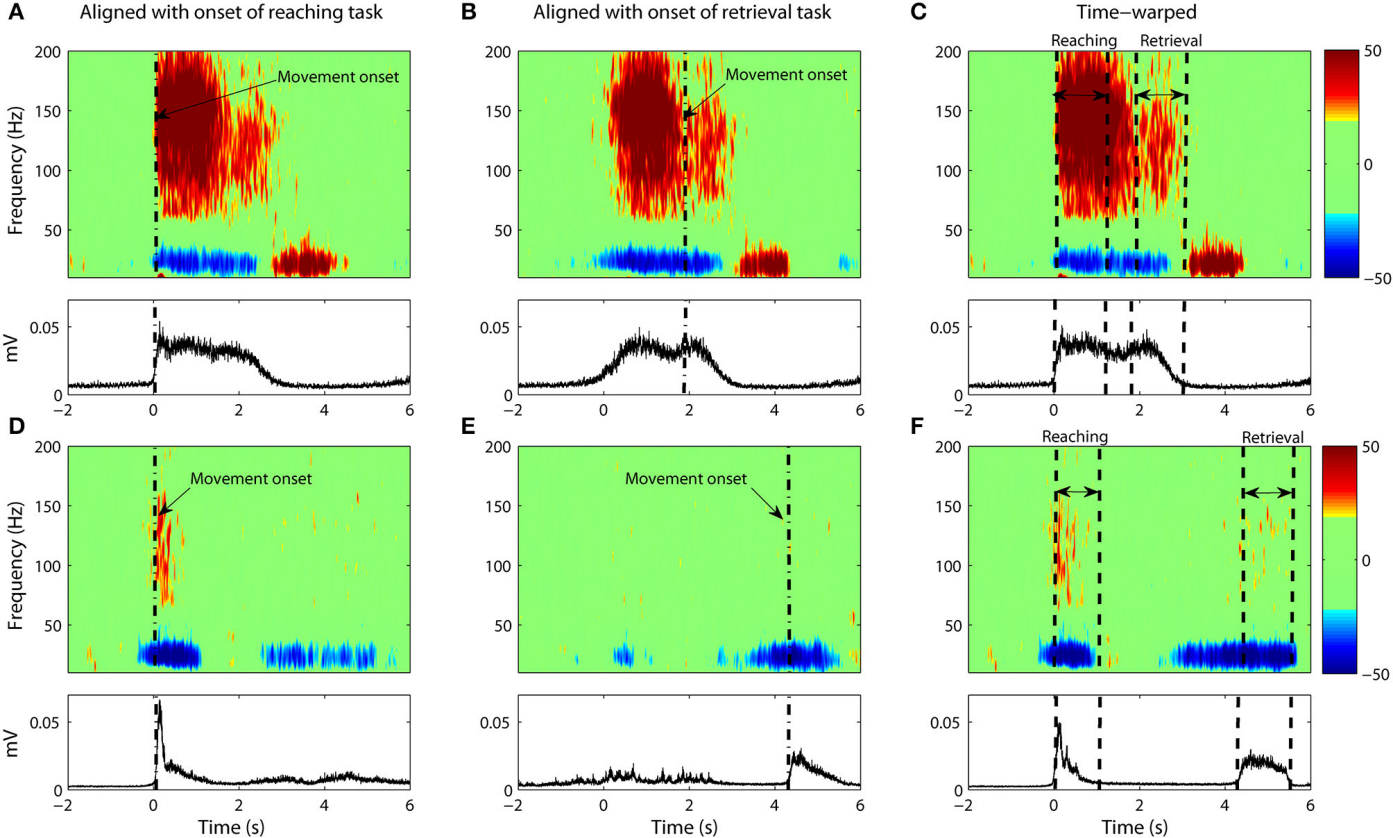
**Same as Figure 4 but with only ECoG data from Subject #3 (A–C) and Subject #4 (D–F)**.

However, the variability in the time course of the velocity profile is significant from trial to trial. Figure 3 shows 25 profiles of arm speed for Subject 1. The movement duration deviated from the average by as much as 600 ms across movement durations that last only 2 s. Spectral components after averaging are visible, but are either blurred or distorted due to temporal misalignment. To illustrate the effects of averaging without non-linear alignment, we carried out our analysis both by aligning to the movement onset of the reach task (Figures 4A,D and Figures 5A,D) as well as aligning to the movement onset of the retrieval task (Figures 4B,E and Figures 5B,E). In each case, gamma activity for example is scattered over the time-course of the movement and localized into multiple components. The breakup of the components is due to temporal misalignment, a result which we also observe with the EMG activities. Also the average of the rectified EMG shows a sharply increasing signal at the onset of the reaching task (Figures 4A and Figures 5A), but as the trials become desynchronized over time they do not exhibit the same sharp decrease at the end of the reaching task, nor is the signal as visible at the onset/completion of the retrieval task. Quantitatively, the EMG signals fall in amplitude by as much as 50% after the completion of the reaching task rendering the activity during the retrieval task almost undetectable. Figures 4B,E and Figures 5B,E show alignment with the onset of the retrieval task but there are similar problems here as well.

### Time-warped averaging of ECoG signals

It is clear that a translational shift in time is insufficient to properly align voluntary movements. Next we explore the results that can be obtained when a non-linear alignment is used. Results are shown in Figures 4C,F and Figures 5C,F, which we compare to the earlier results obtained through conventional averaging of the same trials. The movement-related components (ERD/ERS) are clearly delineated in the warped average spectrograms. Moreover, we see that the time-course of these components matches that of the muscular activity. That is, the gamma ERS and beta ERD appear only when the muscles are activated. Synchronization of the gamma activity is clearly observed for both the reaching and retrieval tasks as single components.

Gamma activity for the rest period between the reaching and retrieval tasks is more complicated. For Subject 4, the activity returns to the baseline during the hold phase of the transition between the reaching and the retrieval tasks. For two of the subjects (Subjects 1 and 2), the activity for the hold phase is reduced, but does not go entirely to the baseline. Nevertheless we observe that time warping produces a much clearer “quiet period” in neural activity when compared to alignment by movement onset only. Subject 3 did not conduct the trials according to the instructions provided. There was no hold period in between the tasks and as such no clear drop in gamma activity was observed.

Statistical analysis shows that variability in the spectrograms was reduced significantly for all subjects (*p* < 1%) with the exception of Subject 1 (*p* < 40%). While variability for Subject 1 was also reduced after warping, it is of interest to investigate why the results differed dramatically for this one subject. When we examined the trajectories for this subject, we noticed that the subject often overshot the target point (this was observed in more than one third of their trials). Since the time-registration path is calculated from the trajectories themselves, we therefore calculated the RMS error from warping the kinematic trajectories alone. What we found was that the error for Subject 1 greatly exceeded the error of the other subjects (by at least a factor of 2).

## Discussion

In this paper, we presented a new method for alignment of neural events over repeated experimental trials. Self-paced unconstrained voluntary movements are prone to movement variability in initiation time, duration and speed. The movement-related cortical activities would therefore occur at different time instances across different trials. These temporal mismatches add to the difficulty in identifying the salient neural response underlying movement activity. Given that there exists a direct functional relationship between neural activity and arm kinematics, we hypothesize that neural events can be better aligned if the kinematic profiles are identical on each trial. Kinematic signals were used to find a non-linear transformation of the time axis as calculated by the method of dynamic time-warping. The transformations serve to remove any temporal variabilities in the way the task was performed. The time transformations were then applied to the spectrogram of the corresponding ECoG signal. This resulted in well-delineated movement-related components including event-related synchronization/desynchronization. Finally, the spectrograms that are now aligned were averaged. The outcome is a vastly improved visualization of the neural activity for complex arm movements. When the results are compared to that of conventional averaging with trials aligned only by movement onset, we see instead the movement-related components to be either blurred or absent. The components found through alignment via warping can be traced back to specific events like movement termination and initiation.

Epochs of EEG data are traditionally aligned with respect to an event of interest (e.g., movement onset). However, if the investigator wishes to study another event in the same data set, the data must be realigned to a new marker. Dynamic time-warping eliminates the need for this as an entire trial is aligned on one go. This has the distinct advantage of allowing for the best possible representation of neural activity across an entire trial. Earlier works have indicated that there is a direct, functional linear relationship between cortical activity and arm velocity (Paninski, 2003; Leuthardt et al., 2004; Chin et al., 2007; Schalk et al., 2007; Pistohl et al., 2008; Bougrain and Liang, 2009; Ganguly et al., 2009; Zhuang et al., 2010). We have made use of this relationship to develop our method. The trials are aligned in accordance with the hypothesis that specific patterns of neural activity (particularly in the beta and gamma activity bands) correspondence linearly with movement velocity. Alignment with velocity would therefore result in the alignment of neural activity.

In choosing relatively well-delineated motor tasks, we did this not because of any limitation in our methodology. So long as the association with velocity holds, the method of temporal alignment should work for more complex tasks (e.g., a tasking involving both reaching and manipulation). However, it is entirely conceivable that our current approach is not complete and that future studies will allow for better methods of temporal alignment involving more complicated movement tasks.

Our findings with ECoG data show a very distinct pattern of activity over the course of the movement. The power in the beta activity (12–30 Hz) was found to be attenuated during the motor tasks whereas power in gamma activity (65–140 Hz) increased correspondingly over the same time period. Beta activity is typically believed to have an inhibitory effect on movement while gamma oscillations are believed to facilitate movement (Pfurtscheller et al., 2003; Jurkiewicz et al., 2006). Activity in both bands return to normal levels after cessation of movement although the time-course for recovery is very different from movement initiation where the neural activity is more abrupt. Similar patterns in neural activity have been found in other recording paradigms: single unit recording, local field, and scalp recordings (Graimann et al., 2003, 2004; Pfurtscheller et al., 2003; Mehring et al., 2004).

One might ask whether it is possible to obtain time-registration paths directly from the spectrograms and to use them to verify the results obtained from kinematics. In theory, it is possible although in practice the neural response is far noisier and thus warping with the spectrogram will not likely result in time-registration paths with the same consistency as the kinematics. Nevertheless, we can attempt such an analysis by restricting warping to only the gamma activity which is relatively clean in our ECoG recordings. Gamma activity is band-passed between 65 and 90 Hz to encompass movement-related gamma activity for all three subjects. We then used gamma to obtain new time-registration paths from which the kinematic signals were then warped. An RMS error is calculated between the warped kinematic signal and the target signal. The RMS error was then compared to the error that would be obtained if only onset alignment was used. We obtained *p*-values of 1%, 13%, and 9% for subjects 1–3 respectively showing results which trend toward significance despite the noisy nature of the neural signals.

We note that subjects with motor impairments often show greater variability in executing motor tasks. As such, the differences between the movement phases can be less marked. In fact, motor coherency studies in the basal ganglia have demonstrated that subjects with Parkinson's disease show more distinctive changes when subjects are on medication rather than off (Cassidy et al., 2002; Levy et al., 2002; Williams et al., 2002; Androulidakis et al., 2007; Jenkinson and Brown, 2011). One might speculate that the disease state introduces variability that would in turn make temporal alignment more difficult. In more extreme cases, where there are imagined but no actual arm movements, our methods would require modification. For such cases, dynamic time warping can be carried out directly on the spectrogram without the use of kinematics. This is typically what happens for speech where one utterance is warped directly into another utterance (Sakoe and Chiba, 1978; Rabiner and Juang, 1993). Although we did not study covert movements, earlier ECoG and fMRI measurements have demonstrated that task-related cortical activity are similar for imagined movements and actual movements (Porro et al., 1996; Graimann et al., 2004; Leuthardt et al., 2004; Shenoy et al., 2008; Miller et al., 2010). For example, Miller et al. (2010) showed that the ECoG gamma response of the motor cortex for imagined movements are initially 25% lower than that of overt movements. However, over time subjects learned to use imagined movements to control a BCI system with induced response eventually exceeding the response of overt movements. Similarly, Leuthardt et al. (2004) showed that modulations of gamma activity are highly correlated with imagined joystick movements. Because of this correlation, one could attempt to warp gamma activity from one imagined movement to another in the manner shown earlier. However, since neural activity is far noisier than the kinematic trajectory, the warping of neural response will be less consistent. Nevertheless, this approach holds promise for movement classification when no kinematic measurement is available.

**Figure 6:**
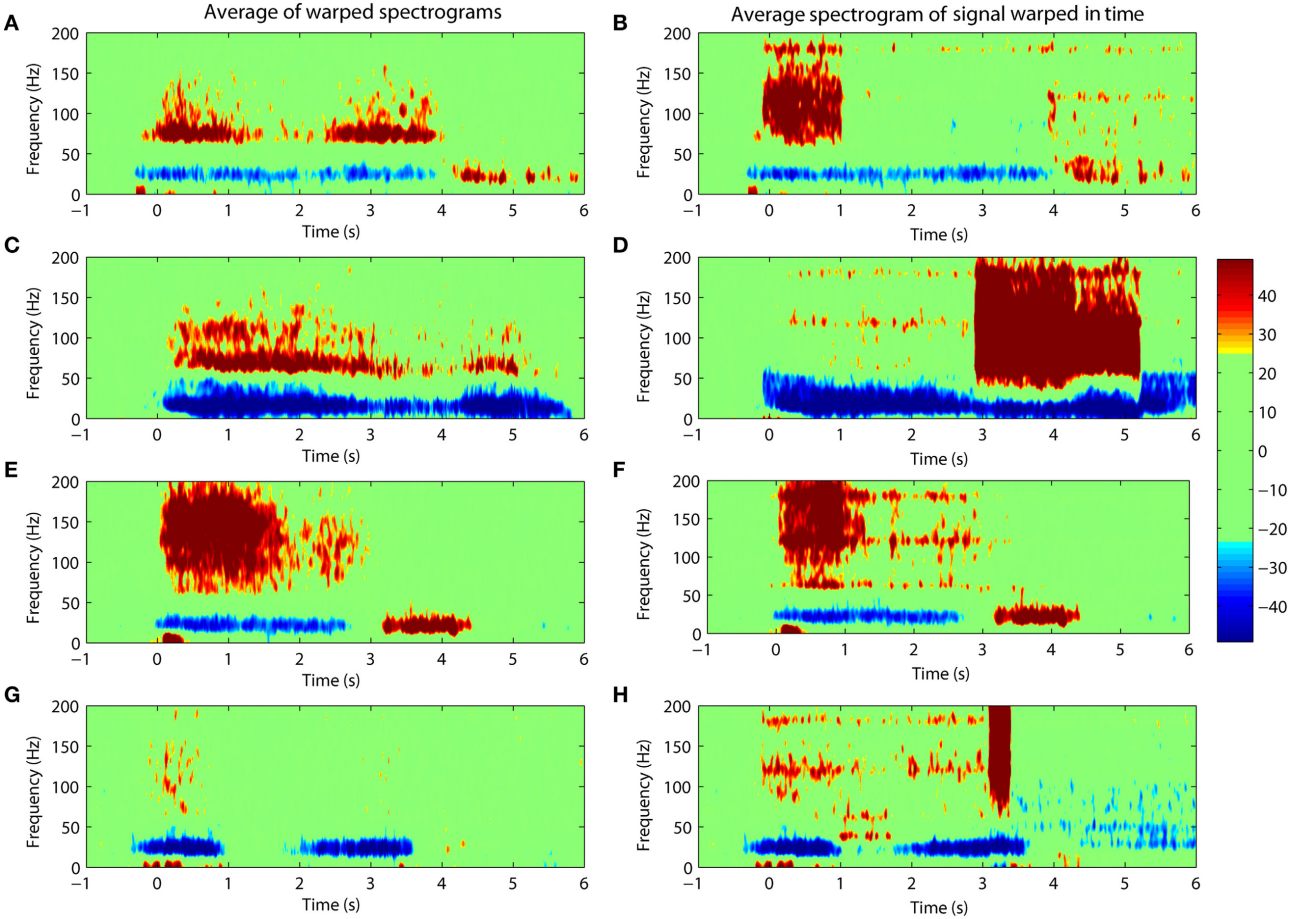
**Comparing the warping of spectrograms (left panels) with the spectrograms of signals warped in the time-domain (right panels)**. Percentile changes in spectral density of the ECoG activity is shown with respect to the rest period. **(A/B,C/D,E/F,G/H)** shows results for Subject #1, #2, #3, #4 respectively. The distortion is evident in the spectrograms after warping is carried out on the time-domain signal.

The results of this study were obtained through the alignment of the spectrograms for cortical activities. An alternative approach is to carry out the alignment directly on the time-domain signal itself. This is not advisable because warping the signal directly distorts its frequency content. Nevertheless, we show an example of what would happen if such an operation were carried out. Warping was applied directly to the ECoG signal followed by a calculation of its spectrogram. Figure 6 compares the averaged spectrogram obtained by this new method with the spectrograms obtained from the original (and preferred) method. We note that the results from warping the time-domain signals are less clear and show obvious distortions due to the prolongation of harmonic signals from line noise as seen in Figures 6B,D,F,H.

There are a number of ways in which a BMI system can decode user intention. One way is to decode single trials by means of, say, movement onset detection followed by a real-time mapping of neural activity onto kinematic movement (Schalk et al., 2007; Pistohl et al., 2008; Wang et al., 2012; Lew et al., 2014; Xu et al., 2014). A second way is to classify neural activity into a number of distinct states or tasks (e.g., rest vs. reaching vs. grasping) (Chin et al., 2007; Pistohl et al., 2012). Our immediate interest is with the latter type of BMI which is more limited but still holds important potential applications. Activity is classified using a database of templates each corresponding to a different task. Our present study is focused on finding better ways to obtain an optimal set of templates through prior time alignment of individual trials before averaging. While we do not consider directly this problem here, it is a simple extension of our methods to allow for classification. This can be done through one of several ways. One way is to remove the reliance on kinematics and to warp directly one neural spectrogram onto another. The score obtained in time-alignment will indicate to which class the movement belongs. One expects that movements drawn from the same class will yield a lower score than warping two movements belonging to different classes (Jeong et al., 2011). A second approach is to develop an optimal series of templates by which any given movement can then be scored by comparing it to a “gold standard.” The highest score then defines the class of movement. Obviously much of our ideas are motivated by the classic work done in speech recognition (Rabiner and Juang, 1993). While most modern implementations of automatic speech recognition systems use statistical models like hidden Markov models or neural networks, the basic principles remain the same. Our choice of dynamic time warping was motivated by the ease of implementation as well as its relevance toward the basic scientific question of finding the underlying neural components of upper limb movements.

### Conflict of interest statement

The authors declare that the research was conducted in the absence of any commercial or financial relationships that could be construed as a potential conflict of interest.
